# Decomposition of Heart Rate Variability Spectrum into a Power-Law Function and a Residual Spectrum

**DOI:** 10.3389/fcvm.2016.00016

**Published:** 2016-06-02

**Authors:** Jane Kuo, Cheng-Deng Kuo

**Affiliations:** ^1^Laboratory of Biophysics, Department of Medical Research, Taipei Veterans General Hospital, Taipei, Taiwan; ^2^Chest Medicine and Physiological Signals Research Center, Changhua Christian Hospital, Changhua City, Taiwan

**Keywords:** heart rate variability, power spectrum, power-law function, fractal, decomposition, slope, *Y*-intercept

## Abstract

The power spectral density (PSD) of heart rate variability (HRV) contains a power-law relationship that can be obtained by plotting the logarithm of PSD against the logarithm of frequency. The PSD of HRV can be decomposed mathematically into a power-law function and a residual HRV (rHRV) spectrum. Almost all rHRV measures are significantly smaller than their corresponding HRV measures except the normalized high-frequency power (nrHFP). The power-law function can be characterized by the slope and *Y*-intercept of linear regression. Almost all HRV measures except the normalized low-frequency power have significant correlations with the *Y*-intercept, while almost all rHRV measures except the total power [residual total power (rTP)] do not. Though some rHRV measures still correlate significantly with the age of the subjects, the rTP, high-frequency power (rHFP), nrHFP, and low-/high-frequency power ratio (rLHR) do not. In conclusion, the clinical significances of rHRV measures might be different from those of traditional HRV measures. The *Y*-intercept might be a better HRV measure for clinical use because it is independent of almost all rHRV measures. The rTP, rHFP, nrHFP, and rLHR might be more suitable for the study of age-independent autonomic nervous modulation of the subjects.

## Introduction

Heart rate variability (HRV) refers to the continuous oscillation of RR intervals (RRIs) around its mean value. Power spectrum analysis of heart rate (HR) fluctuations provides a quantitative and non-invasive means to assess the sympathetic and vagal modulations of HR (1, 2). The imbalance in the two branches of autonomic nervous modulation may contribute to and reflect many kinds of illness. HRV analysis has long been used in the assessment of many diseases such as acute myocardial infarction (AMI) (3, 4), post-myocardial infarction status (5), and orthotopic heart transplantation (6).

The HR is influenced by the complex interplay of neural, humoral, and electrophysiological factors, which in turn are modulated by central and peripheral oscillators (7). To evaluate the complexity of the controlling system of HR, the power-law characteristics of power spectral density (PSD) can be obtained by plotting the logarithm of PSD against the logarithm of frequency (Frq) to obtain a linear regression line (8–16). The existence of the linear regression line indicates that the relationship between PSD and Frq in the power spectrum of HRV can be described by a power-law function. The power-law relationship of HRV has been used as a predictor of mortality in the elderly (9), and the analysis of the fractal characteristics of short-term RRI dynamics can yield more powerful prognostic information than the traditional HRV measures among patients with depressed left ventricular function after AMI (10), patients with Chaga’s disease (15), and pediatric patients with multiple organ failure (16).

Since power-law relationship between PSD and Frq is an important ingredient of HRV spectrum, it is possible to decompose mathematically the HRV spectrum into a power-law relationship between PSD and Frq and a remaining part. Thus, the aims of this study were to decompose the PSD of traditional HRV into a power-law function and a residual HRV (rHRV) and to compare the rHRV measures with their corresponding HRV measures in healthy adults.

## Materials and Methods

### Study Subjects

The healthy subjects were volunteers recruited from the community. The subjects included in this study did not have known cardiopulmonary or other systemic disease, which may influence HRV. This research has been approved by the Ethics Committee of the Taipei Veterans General Hospital. Informed consent was obtained from each subject before the study.

### Physiological Measurements

Sixty healthy subjects recruited from the community participated in this study. The general characteristics of these healthy subjects are shown in Table 1. The study subject was requested to not take caffeinated or alcoholic beverages 24 h prior to the study. After 5 min rest in sitting position, a trend of electrocardiogram (ECG) signals was picked up by a multichannel recorder (Biopac MP150 with 16 channels, BIOPAC Systems, Inc., Goleta, CA, USA) from conventional lead II on each subject. The analog signals of ECG were transformed into digital signals by using an analog-to-digital converter with a sampling rate of 500 Hz. The ECG signals were recorded for 10 min, so that at least 512 inter-beat intervals (RRIs) can be obtained for HRV analysis. All procedures were performed in the afternoon in a bright and quiet room with a room temperature of 24–25°C and humidity of 54–55%.

**Table 1:** **General characteristics and the slope and *Y*-intercept of linear regression of HRV spectra of the study subjects (*n* = 60)**.

Gender (M/F)	23/37
Age (years)	34.9 ± 13.4
Body height (cm)	164.2 ± 8.6
Body weight (kg)	60.2 ± 10.2
Body mass index (kg/m^2^)	22.2 ± 2.6
Systolic blood pressure (mmHg)	115.8 ± 14.8
Diastolic blood pressure (mmHg)	72.9 ± 9.4
Pulse pressure (mmHg)	42.9 ± 10.7
Mean arterial blood pressure (mmHg)	87.2 ± 10.3
Heart rate (bpm)	76.5 ± 9.9
Slope	1.34 ± 0.36
*Y*-intercept	1.39 ± 0.55

### HRV Analysis

The method of HRV analysis has been reported previously (17). In brief, the recorded ECG signals were retrieved to measure the consecutive RRIs, which are the time intervals between successive pairs of QRS complexes, by using the software for the detection of R wave. The atrial or ventricular arrhythmia was deleted before HRV analysis. If the percentage of deletion was >5%, then the data of the patient were excluded from HRV analysis. The last 512 stationary RRI were used for HRV analysis.

The fast Fourier transform (FFT) of Cooley and Tukey expresses the discrete Fourier transform (DFT) of an array of size *N* recursively in terms of two DFTs of size *N*/2 to reduce the overall runtime of computations (18). The basis of the binary form of FFT is the Danielson–Lanczos lemma, which breaks each term in the array again and again into even and odd terms until the samples are exhausted (19). Thus, a binary form of FFT requires the number of points *N* in the array to be a power of 2, or *N* = 2*^*r*^*, where *r* is an integer. In this study, we chose *N* = 2^9^ = 512 stationary RRI for HRV analysis, so that the time required for ECG recording could be <10 min in general.

The mean, SD (SD_RR_), coefficient of variation (CV_RR_), and rMSSD of 512 RRI were calculated using standard formula. The power spectra of the 512 RRI were obtained by means of fast Fourier transformation (Mathcad 15.0, Mathsoft Inc., Cambridge, MA, USA). Direct current component was excluded before the calculation of the powers. The area under the curve of the spectral peaks within the Frq range of 0.01–0.4, 0.01–0.04, 0.04–0.15, and 0.15–0.40 Hz were defined as the total power (TP), very low-frequency power (VLFP), low-frequency power (LFP), and high-frequency power (HFP), respectively.

The Task Force of the European Society of Cardiology and the North American Society of Pacing Electrophysiology (2) has suggested that the power within the Frq range of 0.04–0.4 Hz be used for the normalization of LFP and HFP. Since this Frq range does not cover the Frq ranges of VLFP and may not be suitable for the normalization of VLFP, we used the power within the Frq range of 0.01–0.4 Hz to normalize VLFP, LFP, and HFP in this study. The normalized VLFP (nVLFP = VLFP/TP) was used as the index of vagal withdrawal, renin–angiotensin modulation, and thermoregulation (20–22), the normalized low-frequency power (nLFP = LFP/TP) as the index of combined sympathetic and vagal modulation (23), the normalized HFP (nHFP = HFP/TP) as the index of vagal modulation, and the low-/high-frequency power ratio (LHR = LFP/HFP) as the index of sympathovagal balance (24).

### Mathematical Decomposition of PSD

In the study by Huikuri et al. (9), the power-law scaling of the power spectra (exponent β) from 24-h Holter recordings was calculated from the Frq range of 0.0001–0.01 Hz. This Frq range did not cover the spectral peaks in the very low-frequency (VLF), low-frequency (LF), and high-frequency (HF) ranges. To facilitate the decomposition of the whole HRV spectrum, the power-law relationship of HRV was calculated by plotting log(PSD) against log(Frq) within the Frq range from >0 Hz to the Nyquist Frq in this study. The 0 Hz point must be excluded because log(0) is not defined mathematically.

Since there is a linear relationship between the log(PSD) and log(Frq) in the power spectrum of HRV, the linear regress relationship between log(PSD) and log(Frq) can be expressed as

(1) $$\text{log}\left({\text{PSD}}_{\text{rg}}\right)=\text{s}\times \text{log}\left(\text{Frq}\right)+\text{Y,}$$

where the “log” denotes logarithm, the subscript “rg” stands for “regression,” and the “*s*” and “*Y*” are the slope and *Y*-intercept of linear regression between log(PSD_rg_) and log(Frq) within the Frq range from >0 Hz to the Nyquist Frq, respectively. The PSD_rg_ is the PSD that can be accounted for by the linear regression equation between log(PSD) and log(Frq). Thus, we have

(2) $${\text{PSD}}_{\text{rg}}=10^{\text{s}\times \text{Log}\left(\text{Frq}\right)+\text{Y}}=10^{\text{Y}}\times {\text{Frq}}^{\text{s}}.$$

It is clear that the PSD_rg_ is a power-law function of Frq with scaling exponent *s* and a constant 10^Y^. The difference between PSD and PSD_rg_ is the power-law free PSD or the residual PSD (rPSD) that cannot be accounted for by the power-law function shown in Eq. 2:

(3) $$\text{log}\left(\text{rPSD}\right)=\text{log}\left(\text{PSD}\right)-\text{log}\left({\text{PSD}}_{\text{rg}}\right)=\text{log}\left(\text{PSD}/{\text{PSD}}_{\text{rg}}\right),$$

(4) $$\text{rPSD}=\frac{\text{PSD}}{{\text{PSD}}_{\text{rg}}}=\text{PSD}\times (10^{-\text{Y}}\times {\text{Frq}}^{-\text{s}}).$$

In this way, the PSD can be decomposed into two parts, the PSD_rg_ and the rPSD. The following simple equation depicts the relationship among PSD, PSD_rg_, and rPSD:

(5) $$\text{PSD}={\text{PSD}}_{\text{rg}}\times \text{rPSD}=10^{\text{Y}}\times {\text{Frq}}^{\text{s}}\times \text{rPSD}.$$

### rHRV Measures

Similar to the definition of traditional HRV measures, the area under the curve of the spectral peaks within the range of 0.01–0.4, 0.01–0.04, 0.04–0.15, and 0.15–0.40 Hz in the rPSD were defined as the residual total power (rTP), very low-frequency power (rVLFP), low-frequency power (rLFP), and high-frequency power (rHFP) of the rHRV, respectively. The normalized rVLFP (nrVLFP = rVLFP/rTP), normalized rLFP (nrLFP = rLFP/rTP), normalized high-frequency power (nrHFP = rHFP/rTP), and low-/high-frequency power ratio (rLHR = rLFP/rHFP) were also defined in a similar way to those of HRV measures.

### Statistics

The Wilcoxon signed-rank test (SigmaStat statistical software, SPSS Inc., Chicago, IL, USA) was employed to compare the traditional HRV measures with the corresponding rHRV measures. Linear regression analysis was used to find the relations between the clinical characteristics and the measures of HRV and rHRV. All data are presented as median (25–75%). A *P* < 0.05 was considered statistically significant.

## Results

**Figure 1:**
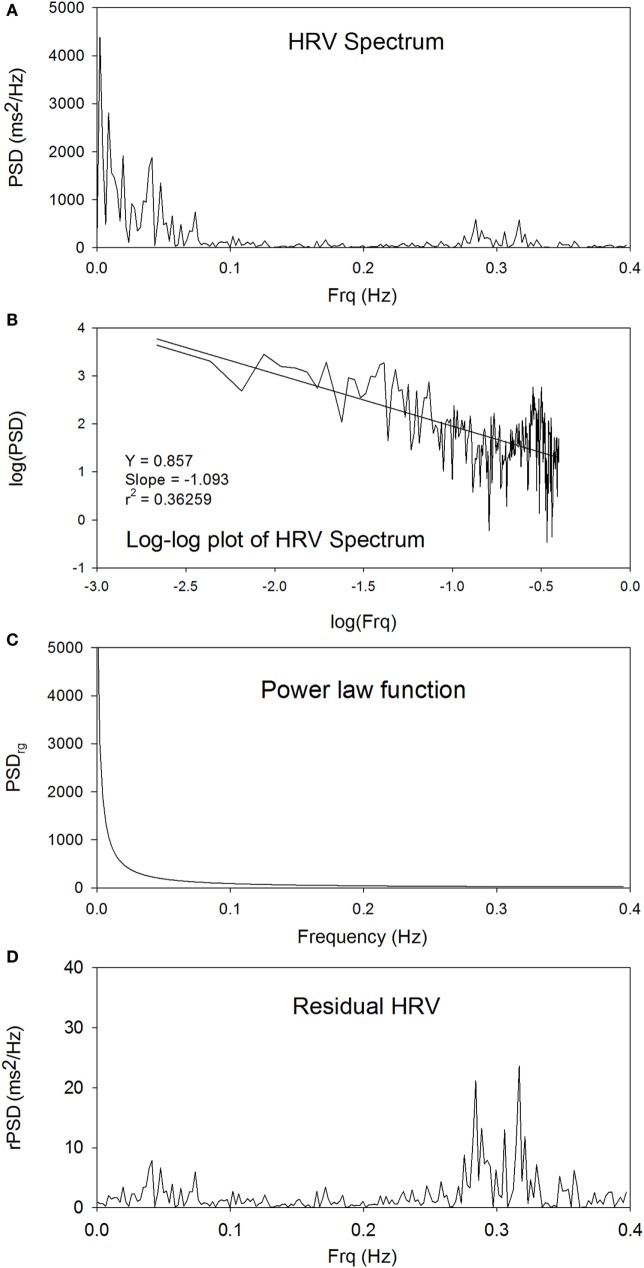
**The power spectrum of traditional HRV (A), the linear plot of log(PSD) versus log(Frq) (B), the power-law function between PSD_rg_ and frequency in the linear plot (C), and the plot of rPSD (D)**.

Figure 1A shows the power spectrum of traditional HRV. The peaks at around 0.3 Hz are the respiratory components. Figure 1B shows the linear plot of log(PSD) versus log(Frq). There is a statistically significant linear relationship between log(PSD) and log(Frq), indicating that an inverse power-law scaling relationship exists between PSD and Frq, and that the power spectrum of HRV is a fractal. The slope and *Y*-intercept can be obtained from linear regression analysis of log(PSD) versus log(Frq) to characterize the power spectrum of HRV. Figure 1C shows the power-law function between PSD_rg_ and Frq in the linear plot. The subscript “rg” denotes “regression.” Figure 1D shows the residual PSD after the removal of the power-law relationship between PSD_rg_ and Frq. The spectral peaks in the low-Frq range are suppressed, whereas the high-Frq peaks at around 0.3 Hz are enhanced, as compared with the original power spectrum shown in Figure 1A.

**Figure 2:**
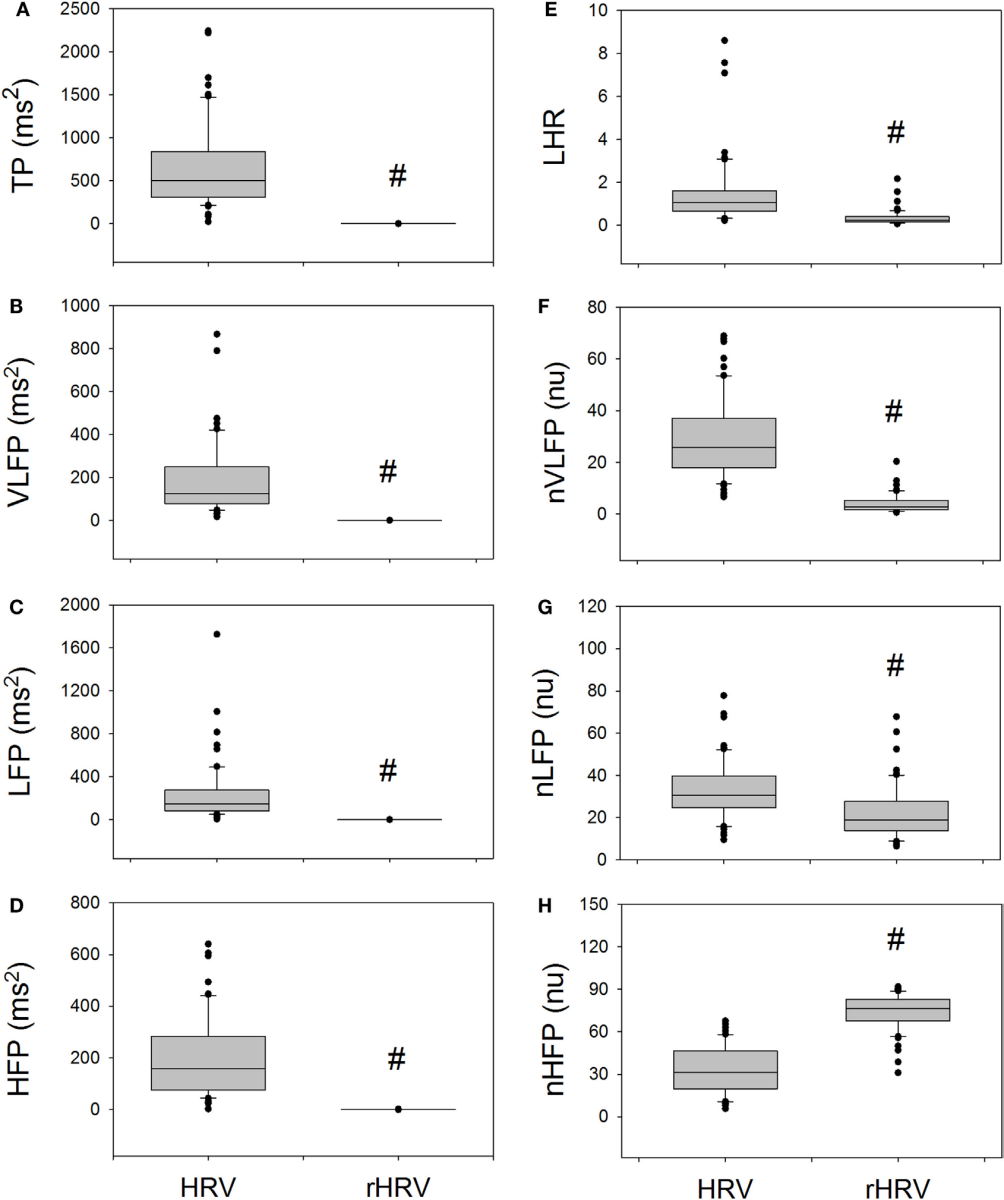
**The comparison of traditional HRV measures with the corresponding rHRV measures**. # indicates statistical significance vs. corresponding HRV measure.

Figure 2 compares the measures of HRV and rHRV. The rTP, rVLFP, rLFP, rHFP, rLHR, nrVLFP, and nrLFP of rHRV are all significantly smaller than their corresponding HRV measures, whereas only the nrHFP is significantly greater than the nHFP. In short, the rHRV has significantly enhanced high-Frq component and significantly suppressed lower Frq components, as compared with traditional HRV. This means that the removal of the power-law function from the traditional HRV can disclose more details of the nrHFP of HRV.

**Figure 3:**
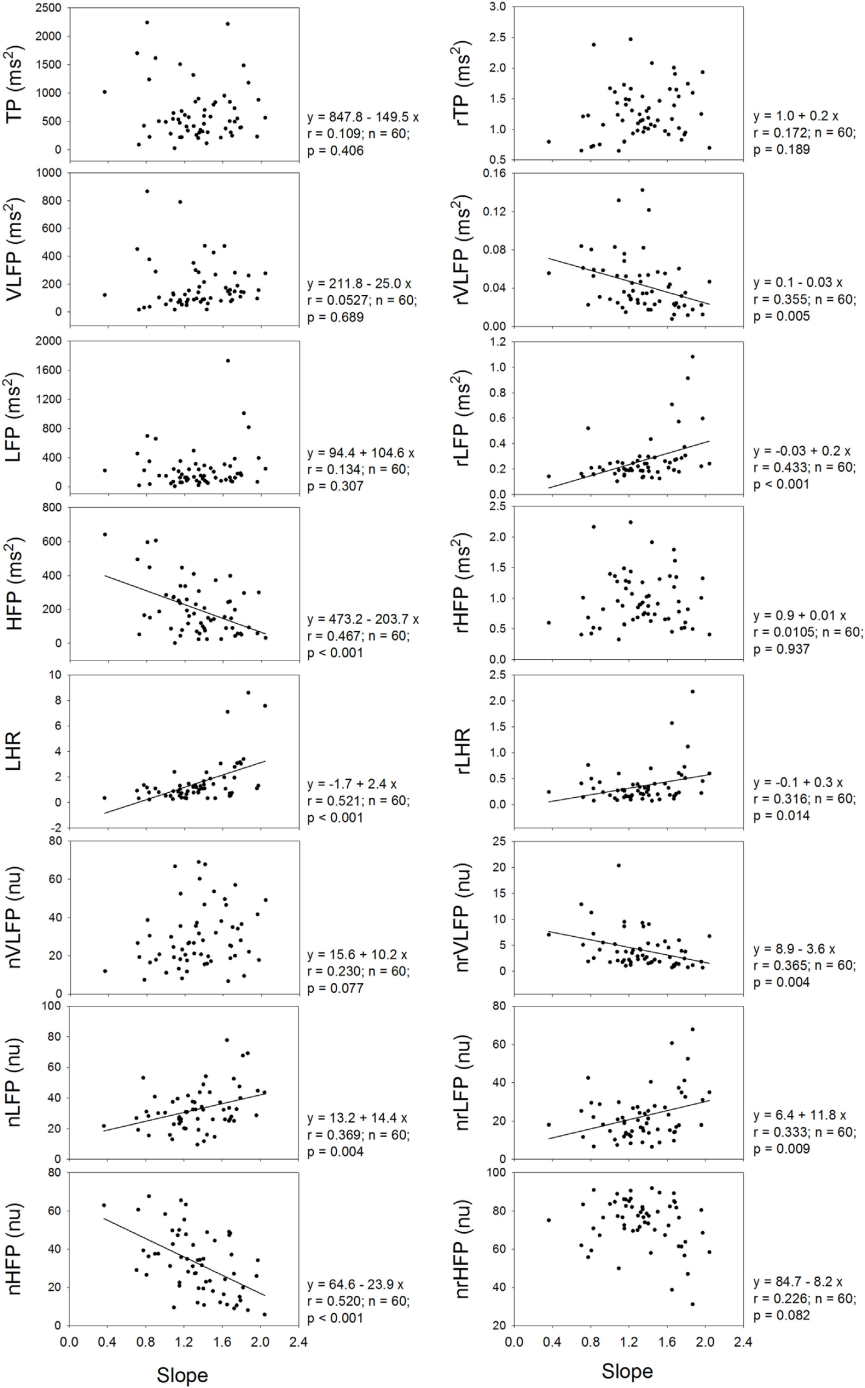
**The dependence of traditional HRV measures and rHRV measures on the slope of linear regression or the exponent of power-law function**.

Figure 3 shows that the exponent or slope of linear regression of the power-law function correlates significantly and positively with the LHR and nLFP of traditional HRV, and significantly and negatively with the HFP and nHFP of traditional HRV. In the rHRV, the exponent still correlates significantly and positively with the rLFP, rLHR, and nrLFP; however, it does not correlate with either HFP or nHFP. Instead, the exponent correlates significantly and negatively with rVLFP and nrVLFP. These results suggest that the removal of the power-law function from the traditional HRV also removes the dependence of high-Frq component of HRV on the exponent, but uncovers the dependence of low-Frq and very low-Frq component of HRV on the exponent instead. That is, the high-Frq component of rHRV has no correlation with the exponent of the power-law function, but the low-Frq and very low-Frq component of rHRV have.

**Figure 4:**
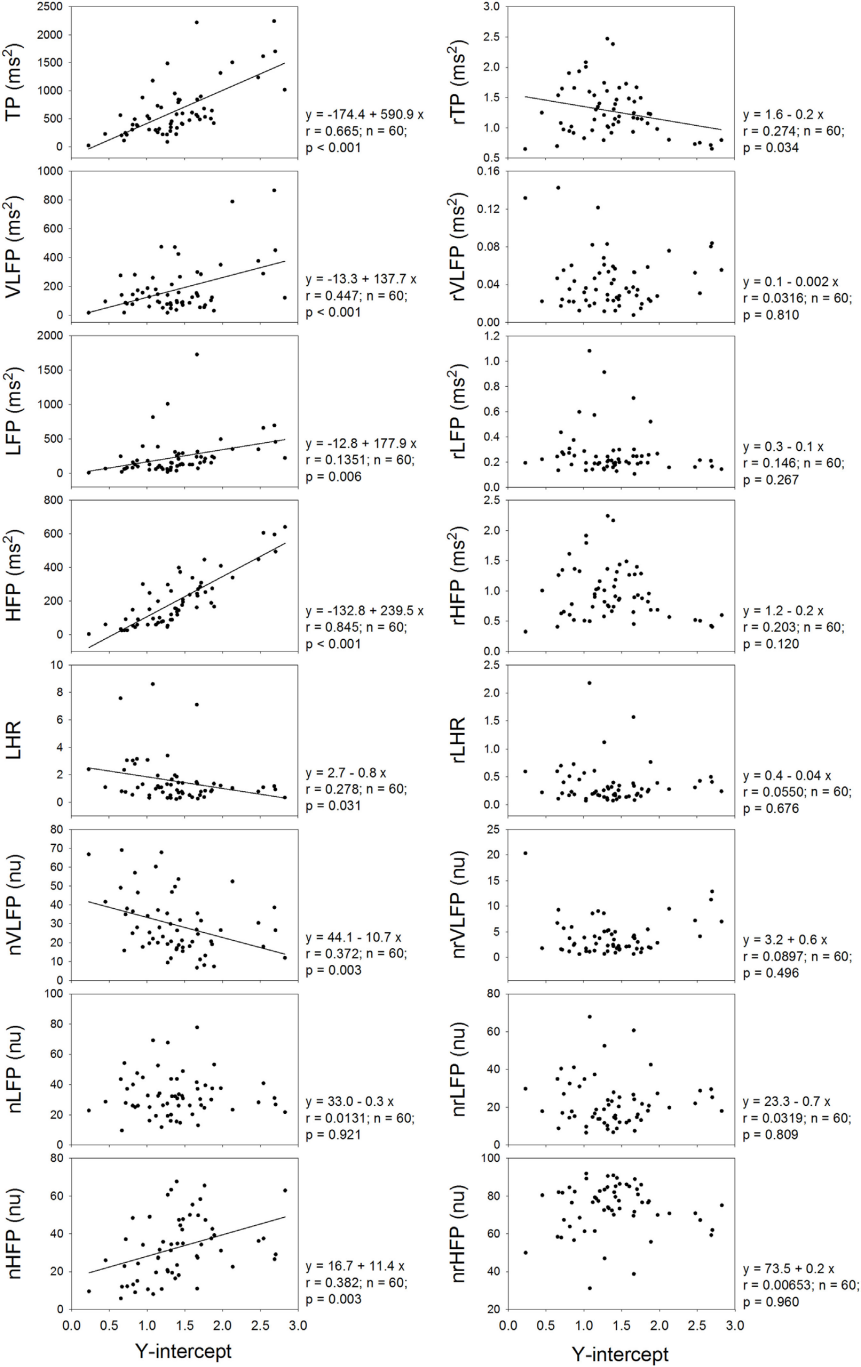
**The dependence of traditional HRV measures and rHRV measures on the *Y*-intercept of linear regression**.

Figure 4 shows that the *Y*-intercept has significant and positive correlations with the TP, VLFP, LFP, HFP, and nHFP of traditional HRV and has significant and negative correlations with LHR and nVLFP. In the rHRV, the *Y*-intercept has significant and negative correlation with rTP only. Even though the *Y*-intercept still has significant correlation with rTP, the correlation is a negative one, rather than a positive one in traditional HRV. It seems that the *Y*-intercept of linear regression is an important ingredient in the traditional HRV because almost all HRV measures have significant correlations with the *Y*-intercept except the nLFP, and because the removal of the power-law function from traditional HRV results in no significant correlations between *Y*-intercept and almost all rHRV measures except the rTP.

**Figure 5:**
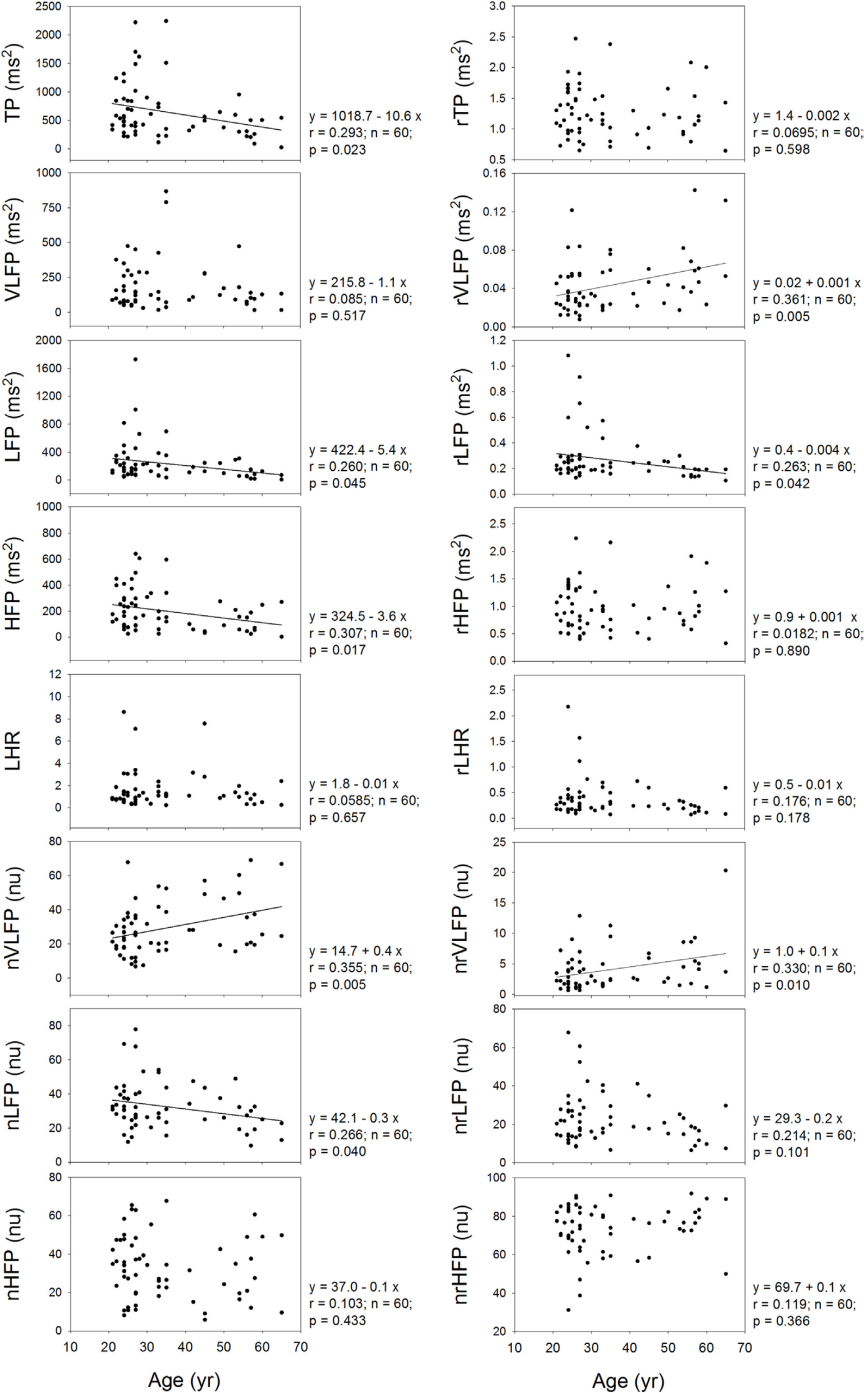
**The dependence of traditional HRV measures and rHRV measures on the age of the subjects**.

It is already known that the HRV measures depend on the age of the subjects. Therefore, it is necessary to inquire whether or not the rHRV measures still depend on the age of the subjects. Figure 5 shows that the TP, LFP, HFP, nVLFP, and nLFP of traditional HRV depend on age. In the rHRV, the age still correlates significantly with the rVLFP, rLFP, and nrVLFP; however, the rTP, rHFP, nrHFP, and rLHR do not depend on age. The non-dependence of rTP, rHFP, nrHFP, and rLHR on age may make them more suitable for the study of age-independent autonomic nervous modulation of the subjects.

## Discussion

This study decomposed the traditional HRV spectrum into a power-law function and an rHRV spectrum and compared the rHRV measures with traditional HRV measures. We found that the rHRV measures are significantly smaller than their counterparts in the traditional HRV except the nrHFP, which is significantly greater than the nHFP in the traditional HRV. The exponent of power-law function does not have any correlation with either HFP or nHFP, but correlate significantly and positively with the rLFP, rLHR, and nrLFP. Although the *Y*-intercept of linear regression has significant correlations with many measures in traditional HRV, it has no correlations with almost all rHRV measures except rTP. Furthermore, although the TP of traditional HRV decreases with increasing age, the rTP of rHRV does not depend on the age of the subject. Since the nrHFP is significantly enhanced as compared to the nHFP in the traditional HRV, whereas other rHRV measures are significantly suppressed as compared to their counterparts in the traditional HRV, the rHRV might be better suited for the study involving the high-Frq component of HRV.

It is well known that the HF components of HRV do not fit the power-law profile. This is illustrated in Figure 1. On this basis, there is no surprise in finding the important residual power in the HF band after the removal of the 1/*f* component. In addition, it is not surprising to find an enhanced nrHFP in the rHRV because the powers of residual lower Frq components are reduced after the decomposition.

In some studies, the Frq range for the power-law relationship in the power spectrum of HRV from the entire 24-h Holter recordings was calculated by using a line-fitting algorithm of log(power) versus log(Frq) within the Frq range of 0.0001–0.01 Hz to yield the scaling exponent (8–10, 13, 14). Other Frq ranges for the calculation of scaling exponent, such as 0.001–1 Hz (11), 0.003–0.1 Hz (12), and 0.0001–0.1 Hz (15), have also been used by various study groups. In this study, the ECG recording was performed for 10 min and a short-term HRV was obtained from 512 RRIs. So long as a power-law relationship exists between the PSD and the Frq, the power spectrum can be decomposed into a power-law function and a residual part no matter how long the ECG recording is. In other word, the decomposition method shown in this study can be used to decompose the power spectrum of HRV for both short-term and long-term HRV, so long as a power-law relationship exists between the PSD and the Frq. If only the PSD within the Frq range of 0.0001–0.01, 0.001–1, 0.0001–0.1, or 0.003–0.1 Hz is going to be analyzed by using the new method, the power spectrum of HRV can also be decomposed within that small Frq range. If, however, the power spectrum within a larger Frq range is going to be analyzed by using this method, then a larger Frq range must be adopted. The size of the Frq range is not a problem for the new method.

The rHRV is obtained by removing the power-law function. It can be expected that the magnitude and clinical significance of rHRV measures are different from their corresponding HRV measures. For instance, the rTP, rVLFP, rLFP, rHFP, nrVLFP, nrLFP, and rLHR are all significantly smaller than their corresponding HRV measures, whereas only the nrHFP is significantly greater than the nHFP. In addition, the dependences of TP, HFP, and nLFP on age in the HRV no longer exist in the rHRV, because the rTP, rHFP, and nrLFP do not depend on age. The dependence of TP, HFP, and nLFP on age might come from the *Y*-intercept because the *Y*-intercept has significant and positive correlations with the TP, VLFP, LFP, HFP, and nHFP and has significant and negative correlations with LHR and nVLFP.

It has been shown that the VLFP of HRV is a powerful predictor of clinical prognosis in patients with congestive heart failure (25). This prognostic value of VLFP of traditional HRV might originate from the *Y*-intercept of the power-law function because there is a significant and positive correlation between VLFP and *Y*-intercept and significant and negative correlation between nVLFP and *Y*-intercept in this study (Figure 4). Since there are no correlations between rVLFP and *Y*-intercept, and between nrVLFP and *Y*-intercept, the rVLFP and nrVLFP might no longer be used as the predictors in patients with congestive heart failure and other diseases. Instead, the *Y*-intercept might have the potential of being used as the predictor in patients with various kinds of diseases.

The rTP, rVLFP, rLFP, rHFP, rLHR, nrVLFP, and nrLFP of rHRV are all significantly smaller than their corresponding HRV measures, while the nrHFP is significantly greater than the nHFP (Figure 2). This finding suggests that the clinical meaning and significance of rHRV measures might be different from traditional HRV measures and that the nrHFP might be a better index of vagal modulation of the subjects. The dependence of almost all HRV measures on the *Y*-intercept and the non-dependence of almost all rHRV measures on the *Y*-intercept (Figure 4) further suggest that the currently used HRV measures are not independent of one another and that the *Y*-intercept and rHRV measures might be better indices of autonomic nervous modulation of the subjects. Further studies are needed to explore the clinical significance and applicability of the *Y*-intercept, slope, and rHRV measures introduced in this study.

## Conclusion

The PSD of traditional HRV can be decomposed into a power-law function displaying the fractal characteristics of the PSD and an rHRV. The decomposition of traditional HRV into a power-law function and an rHRV suggests that the rHRV measures and the exponent and *Y*-intercept of linear regression must be included in future spectral HRV analysis. The clinical significances of rHRV measures might be different from those of traditional HRV measures. The *Y*-intercept might be an important HRV measure for clinical use because almost all rHRV measures have no significant correlation with it. The non-dependence of rTP, rHFP, nrHFP, and rLHR on the age of the subjects might make them more suitable for the study of age-independent autonomic nervous modulation of the subjects.

## Author Contributions

JK: data analysis, statistical analysis, and figures preparation; C-DK: conception, design, and drafting of the work.

## Conflict of Interest Statement

The authors declare that the research was conducted in the absence of any commercial or financial relationships that could be construed as a potential conflict of interest.
